# High Levels of Receptor Tyrosine Kinases in CCM3-Deficient Cells Increase Their Susceptibility to Tyrosine Kinase Inhibition

**DOI:** 10.3390/biomedicines8120624

**Published:** 2020-12-17

**Authors:** Miriam Sartages, Ebel Floridia, Mar García-Colomer, Cristina Iglesias, Manuel Macía, Patricia Peñas, Pierre-Olivier Couraud, Ignacio A. Romero, Babette Weksler, Celia M. Pombo, Juan Zalvide

**Affiliations:** 1 Department of Physiology, Centro Singular de Medicina Molecular e Enfermedades Crónicas (CiMUS), Instituto Sanitario de Santiago de Compostela (IDIS), Universidade de Santiago de Compostela, 15703 Santiago de Compostela, Spain; miriam.sartages@usc.es (M.S.); ebel.floridia@gmail.com (E.F.); m.garcia.colomer@usc.es (M.G.-C.); cristina.iglesias@usc.es (C.I.); celiamaria.pombo@usc.es (C.M.P.); 2 IQVIA RDS Ireland Limited, Eastpoint Business Park, Estuary House, Fairview, Dublin 3, D03 K7W7 Leinster, Ireland; 3 Servicio de Obstetricia y Ginecología Hospital Clínico Universitario Santiago, 15703 Santiago de Compostela, Spain; manuel.macia.cortinas@sergas.es (M.M.); patricia.penas.silva@sergas.es (P.P.); 4 INSERM, U1016, Institut Cochin, 22 Rue Mechain, 75014 Paris, France; pierre-olivier.couraud@inserm.fr; 5 Department of Life, Health and Chemical Sciences, The Open University, Milton Keynes MK7 6AA, UK; nacho.romero@open.ac.uk; 6 Weill Medical College, Cornell University, 1300 York Ave, New York, NY 10065, USA; babette@med.cornell.edu

**Keywords:** cavernoma, CCM3, PDCD10, EGFR, VEGFR2, ErbB2, Lapatinib

## Abstract

Cerebral cavernous malformations (CCMs) are vascular malformations that can be the result of the deficiency of one of the CCM genes. Their only present treatment is surgical removal, which is not always possible, and an alternative pharmacological strategy to eliminate them is actively sought. We have studied the effect of the lack of one of the CCM genes, CCM3, in endothelial and non-endothelial cells. By comparing protein expression in control and CCM3-silenced cells, we found that the levels of the Epidermal Growth Factor Receptor (EGFR) are higher in CCM3-deficient cells, which adds to the known upregulation of Vascular Endothelial Growth Factor Receptor 2 (VEGFR2) in these cells. Whereas VEGFR2 is upregulated at the mRNA level, EGFR has a prolonged half-life. Inhibition of EGFR family members in CCM3-deficient cells does not revert the known cellular effects of lack of CCM genes, but it induces significantly more apoptosis in CCM3-deficient cells than in control cells. We propose that the susceptibility to tyrosine kinase inhibitors of CCM3-deficient cells can be harnessed to kill the abnormal cells of these lesions and thus treat CCMs pharmacologically.

## 1. Introduction

Cerebral cavernous malformations (CCMs, OMIM#116860) are vascular malformations with a high prevalence of 1 in 200–250 individuals. They develop in the venous-capillary vascular bed almost exclusively in the central nervous system, and it is because of this location that they give rise to clinically relevant symptoms, from headaches to seizures and bleeding [1,2,3]. CCMs are low-flow vascular malformations with thrombosis and calcifications as typical features, and their endothelium has altered cell-to-cell junctions, which causes leakiness [4]. These abnormal endothelial features have lent support to the idea that CCMs arise from an abnormal endothelial behavior, which has been confirmed by recent studies [3].

Both sporadic and familial forms of CCMs have been described. Familial CCM (fCCM) is characterized by the presence of multiple lesions and an autosomal dominant inheritance with very high penetrability. There have been three CCM genes identified: CCM1 (KRIT1), CCM2 (MGC4607, OSM, Malcavernin), and CCM3 (PDCD10, TFAR15) [5,6,7]. Mutations in these genes are inherited in heterozygosis, and cavernomas are formed when they are completely inactivated in an endothelial cell because of loss of the non-mutated allele [8,9,10]. CCM genes codify for adaptor proteins [11]. Study of the consequences of the loss of CCM genes in endothelial cells in recent years have shed light on the pathogenic mechanisms of CCMs and have even given rise to proposed pharmacological treatments for these malformations (see below). However, we still do not fully understand the behavior of a CCM cell and thus, how a cavernoma develops and especially how it could be limited or, ideally, deleted.

CCM gene products play roles in many important general cellular functions, such as proliferation and death; adhesion, permeability, cytoskeletal regulation and polarity, ROS homeostasis, transmembrane signaling, membrane handling, organelle homeostasis and secretion, kinase and gene regulation (most importantly the Mitogen-activated protein kinase kinase kinase 3 (MEKK3)/Mitogen-activated protein kinase kinase 5 (MEK5)/ Extracellular signal-regulated protein kinase 5 (ERK5)/Myocyte-specific enhancer factor 2C (MEF2C)/ Kruppel Like Factor 2 and 4 (2–4) axis), and differentiation (for reviews in recent years see [3,12,13,14,15]). Some of these effects have been related to the endothelial phenotype of CCMs, and have inspired therapeutic approaches, several of which are active in preventing lesion development in animal models. The anti-inflammatory drug sulindac inhibits the enhanced β-catenin activity of CCM3-deficient endothelial cells and reduces lesion development in a mouse model of CCM3 deficiency [16]. The Rock inhibitor Fasudil precludes the development of lesions in a chronic model of cavernoma development [17]. 3TSR, an antiangiogenic fragment of the thrombospondin-1, suppresses the development of cavernomas in a CCM1-deficient mouse model [18]. The MEKK3 inhibitor ponatinib can prevent the formation of CCM lesions and reduce the growth of lesions in animal models of cavernoma development [19]. Furthermore, indirubin 3 monoxime, a drug derived from Chinese traditional medicine, alleviates CCM burden in mice [20]. However, to the best of our knowledge none of these treatments has been shown to kill cells deficient of CCM genes, and this would be very useful for people who are diagnosed when lesions are already there.

We show in this article that CCM3 deficiency results in high expression of the Epidermal Growth Factor Receptor (EGFR) and an enhanced susceptibility to inhibition of the EGFR family of proteins. This may give rise to new therapeutic strategies. The strategy we propose would actively kill the abnormal cells in cavernoma lesions. Although animal experiments will be needed to discard possible deleterious effects, it would effectively delete lesions already formed.

## 2. Experimental Section

### 2.1. Cell Culture

Human umbilical vein endothelial cells (HuVECs) were isolated as previously described [21] from umbilical cords obtained from the University Clinical Hospital of Santiago de Compostela where written informed consent was obtained. The procedure was approved by the Hospital ethics committee (Comité de ética da Investigación de Santiago-Lugo, Galicia, Spain), ref 2014/493. HUVECs were routinely grown in VascuLife EnGS Endothelial Cell Culture Medium (LifeLine Cell Technology, Oceanside, CA, USA #LL-0002) supplemented with VascuLife^®^ EnGS LifeFactors^®^ Kit (LifeLine Cell Technology, Oceanside, CA, USA #LS-1019) and 100 units/mL penicillin and 100 µg/mL streptomycin (Gibco Thermo Fisher Scientific, Waltham, MA, USA #15140163). HUVEC between P2 and P4 were used for all experiments.

The A549 human lung adenocarcinoma cell line was obtained from the European Collection of Authenticated Cell Cultures (ECACC, Public Health England, UK). It was maintained in DMEM (Dulbecco’s modified Eagle’s medium) (Sigma, St Louis, MO, USA #D6429) supplemented with 10% fetal bovine serum (FBS) (Gibco Thermo Fisher Scientific Inc., Waltham, MA, USA #10270-106), 100 units/mL penicillin and 100 µg/mL streptomycin (Gibco Thermo Fisher Scientific Inc., Waltham, MA, USA #15140-163).

Human Cerebral Microvascular Endothelial Cells (hCMEC/D3) [22] were obtained from Dr Pierre-Olivier Courad and cultured as recommended, on 0.1% gelatin (Sigma, St Louis, MO, USA #G9391) coated plates in Endothelial Cell Growth Basal Medium-2 (EBM-2, Lonza, Basel, Switzerland #CC-3156) supplemented with 5% FBS (Gibco Thermo Fisher Scientific Inc., Waltham, MA, USA #10270-106), 1.4 μM hydrocortisone (Sigma, St Louis, MO, USA #H2270), 5 µg/mL ascorbic acid (Sigma, St Louis, MO, USA #A4403), 1% chemically defined lipid concentrate (Gibco Thermo Fisher Scientific Inc., Waltham, MA, USA #11905-031), 10 mM 4-(2-hydroxyethyl-1-piperazineethanesulfonic acid (HEPES, Sigma, St Louis, MO, USA #H3375), 1 ng /mL human basic fibroblast growth factor (Gibco Thermo Fisher Scientific Inc., Waltham, MA, USA #13256-029) and 100 units/mL penicillin and 100 µg/mL streptomycin (Gibco Thermo Fisher Scientific Inc., Waltham, MA, USA#15140163). hCMEC/D3 cells between P13 and P26 were used for all experiments.

Cells were transduced with lentiviral particles encoding either of two different human small hairpin RNAs (shRNAs) for CCM3 (shCCM3 and shCCM3.4), or non-targeting (shNT). Oligonucleotides were inserted in pLKO.1 plasmid and lentiviral particles were obtained by its cotransfection with psPAX2 and pMD2.G plasmids in HEK293 cells (oligonucleotides sequences shown in Table S1). All cells were selected with Puromycin (Calbiochem, Merck, Darmstadt, Germany #CAS 58-58-2) after transduction.

### 2.2. Reverse Transcription-Quantitative Polymerase Chain Reaction (RT-qPCR) Analysis

TRIzol reagent (Life Technologies, Carlsbad, CA, USA, #15596018) was used to extract the total RNA from cells, RNA was reverse-transcribed using random hexamers, and the mRNA expression level was determined using PowerUp™ SYBR™ Green Master Mix (Thermo Fisher Scientific Inc., Waltham, MA, USA #A25778) using a StepOnePlus™ Real-Time PCR System.

### 2.3. Antibodies and Drugs

The antibodies used are listed in Table S3.

Lapatinib (Selleckchem, TX, USA #S2111), Vatalanib (Selleckchem, TX, USA #S1101), BIX02189 (Selleckchem, TX, USA #S1531), Chloroquine (Sigma, St Louis, MO, USA #C6628), and MG132 (Sigma, St Louis, MO, USA #C2211) were dissolved in dimethylsulfoxide (DMSO). Vascular Endothelial Growth Factor (VEGF, Sigma, St Louis, MO, USA #V5765) and Epidermal Growth Factor (EGF, Calbiochem, Merck, Darmstadt, Germany #PHG0313) were dissolved in water.

### 2.4. Proteome Analysis

Proteome analysis of HuVECs was performed using the Proteome Profiler Human Soluble Receptor Array Non-Hematopoietic Panel (R&D Systems, MN, USA #ARY012) following manufacturer’s instructions and quantified using the ImageJ software (version 1.52s, National Institute of Health, Bethesda, MD, USA).

### 2.5. Western Blot

Cells were solubilized in cold radioimmunoprecipitation assay (RIPA, receipt by request) lysis buffer plus protease and phosphatase inhibitors. PVDF membranes were blocked for 1  h with TBS/0.1%-Tween buffer containing 5% (*w/v*) non-fatty milk and incubated with the corresponding primary and secondary antibodies, blots were developed using ECL^®^ Chemiluminescent detection reagents (Thermo Fisher Scientific Inc., Waltham, MA, USA #32106) and Fuji Medical X-ray Films (Fujifilm, Shangai, Japan). The software ImageJ (version 1.52s, National Institute of Health, Bethesda, MD, USA) was used to quantify the Western blot signals.

### 2.6. Immunofluorescence

Cells were grown to confluence on poly-l-lysine-coated coverslips (Sigma, St Louis, MO, USA #P4832) and the indicated treatments were performed. Then, cover slips were washed twice with PBS, and fixed for 15 min at room temperature with 4% p-formaldehyde in PBS pH 7.4, at dark conditions, permeabilized with Triton X-100 0.5%, blocked with 10% FBS for 60 min and incubated with indicated primary antibodies overnight at 4 °C, then for 1 h at room temperature with Alexa Fluor^®^ 546 conjugated secondary antibodies, and stained with DAPI. Slides were observed in a confocal microscope (Leica Microsystems, Mannheim, Germany) using a HCX PL APO CS 63x objective. Images were captured and analyzed using the Leica LAS AF software (version 3.7.0.20979, Leica Microsystems, Wetzlar, Germany).

### 2.7. Flow Cytometry

A549 cells were grown to confluence. Then, cells were harvested and fixed for 15 min at room temperature with 4% p-formaldehyde in PBS pH 7.4 in the dark, and incubated with antibody against EGFR (clone EGFR1, #ab30, Abcam, Cambridge, UK) for 1 h at room temperature and for 30 min at room temperature with Alexa Fluor^®^ 488 conjugated secondary antibody, both antibodies in 1% BSA solution. Cells (10^4^ per sample) were acquired in a BD Accuri C6 (Becton Dickinson, Oxford, UK) flow cytometer. Cells were acquired and gated by FSC and SSC and analyzed with FlowJo™ software (version 10.1, FlowJo LLC, Ashland, OR, USA).

### 2.8. Fluorescence Detection of Caspase 3/7 Activation

hCMEC/D3 or HuVEC cells were seeded at 9 × 10^4^ cells/well in 24-well plates. The following day, 2 µM Lapatinib was added alongside with 8 μM of CellEvent™ Caspase-3/7 Green Detection Reagent (Invitrogen™, Thermo Fisher Scientific Inc., Waltham, MA, USA #C10423). Cells were imaged by fluorescence at the indicated times after treatment. Apoptotic cells were counted using the ImageJ software (version 1.52s, National Institute of Health, USA).

### 2.9. Statistics

Statistical analysis was performed using GraphPad software (https://www.graphpad.com/), (version 7.0, San Diego, CA, USA) applying the Mann–Whitney test when two groups were compared and ANOVA analysis with a Bonferroni post hoc correction when comparing more than two groups. A value of *p* < 0.05 was considered significant. All graphs represent the mean ± SEM.

## 3. Results

**Figure 1:**
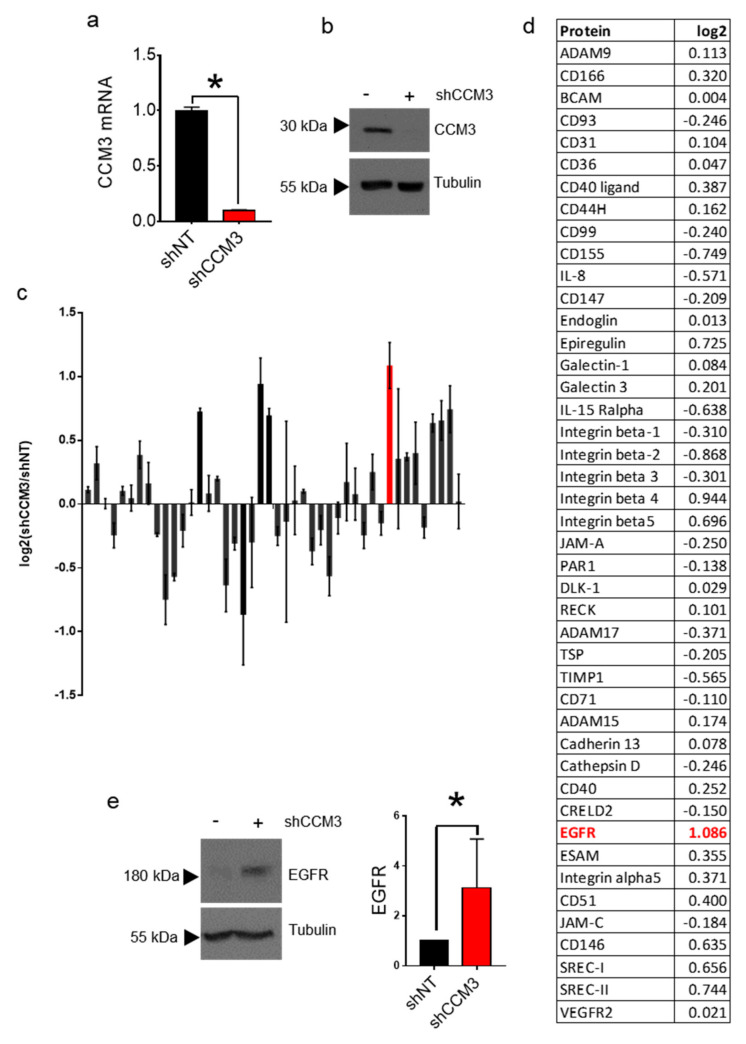
Analysis of membrane proteins in CCM3-deficient human umbilical vein endothelial cells (HUVECs); (**a**) CCM3 mRNA levels in HuVECs transduced with shNT and shCCM3 shRNAs. * *p* < 0.001; (**b**) CCM3 protein levels in the same cells; (**c**) Differences levels of membrane proteins between shNT and shCCM3 HuVECs as seen in a proteome profiler array; (**d**) List of proteins in the proteome profiler assay, with the log2 of their difference between shCCM3 and shNT cells. EGFR shown in red. (**e**) Western blot of Epidermal Growth Factor Receptor (EGFR) HuVECs transduced with shNT and shCCM3 shRNAs. Right panel: quantification of EGFR Western blots. * *p* < 0.05.

To try to identify differences induced by CCM3 deficiency at the protein level, we generated CCM3-deficient HuVECs. CCM3 downregulation using shCCM3 shRNA was efficient at the mRNA (Figure 1a) and protein level (Figure 1b). We then compared the levels of membrane proteins in HuVECs transduced with shNT and shCCM3-expressing lentivirus by using a proteome profiler array. The only protein that had a double intensity on shCCM3 cells was EGFR, whereas several beta-integrins were close to that difference (Figure 1c,d). We were intrigued by the differences in EGFR, as it is a druggable protein with well-developed inhibitors. Furthermore, the levels of the major tyrosine kinase receptor in endothelial cells, VEGFR2, have been proposed to be affected by CCM deletion [23,24,25], but to our knowledge nothing has been seen regarding the EGF receptor.

**Figure 2:**
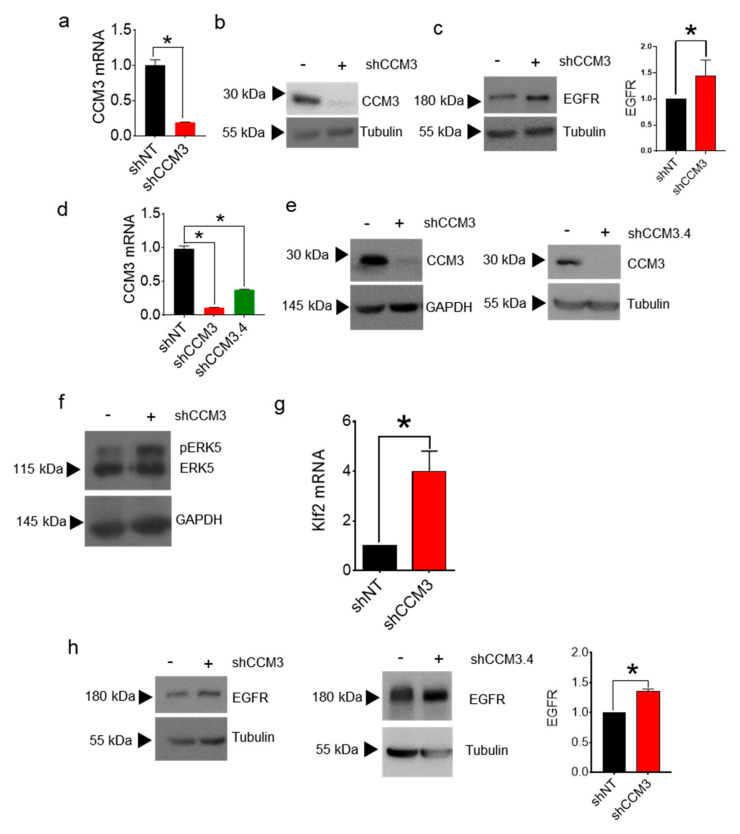
Receptors of the EGFR family are upregulated in several CCM3-deficient cells; (**a**) mRNA levels, and (**b**) Protein levels, of CCM3 in in hCMEC/D3 endothelial cells transduced with shNT and shCCM3 shRNAs. * *p* < 0.001; (**c**) Western blot of EGFR in hCMEC/D3 cells transduced as in (**a**). Right panel: quantification of EGFR Western blots. * *p* < 0.05; (**d**) mRNA levels; and (**e**) Protein levels, of CCM3 in A549 cells transduced with shNT, shCCM3, or shCCM3.4 shRNAs; (**f**) Western blot of ERK5 in A549 cells transduced with shNT or shCCM3 shRNAs; (**g**) mRNA levels of KLF2 in cells transduced as in (**f**); (**h**) Western blot of EGFR in A549 cells transduced with shNT, shCCM3 or shCCM3.4 shRNAs. Right panel: quantification of EGFR Western blots. * *p* < 0.05.

We next looked at the levels of EGFR by Western blot. In accordance to the results of the proteome assay, EGFR was upregulated in CCM3-deficient HuVECs (Figure 1d). Importantly, when we downregulated CCM3 by lentiviral delivery of shRNA in the immortal endothelial cell line hCMEC/D3, a model of blood brain barrier (Figure 2a,b), EGFR was also upregulated (Figure 2c), showing that this effect is not restricted to a particular type of endothelial cell.

To see if the effect of CCM3 on EGFR family proteins could be seen also in cells of non-endothelial origin, we inhibited CCM3 in A549 human adenocarcinoma cells by lentiviral transduction of two different shRNAs. Inhibition of CCM3 was efficient in these cells, both at mRNA (Figure 2d), and protein (Figure 2e) levels with both shRNAs. Interestingly, transduction with shCCM3 showed at least some of the same effects that have been already shown in endothelial cells [3], as seen by hyperphosphorylation of ERK5 (Figure 2f), and stimulation of KLF2 expression (Figure 2g). EGFR was overexpressed in A549 cells transduced with both shCCM3 and shCCM3.4 shRNAs (Figure 2h), suggesting that the role of CCM3 on EGFR regulation is general and not restricted to one cell type.

**Figure 3:**
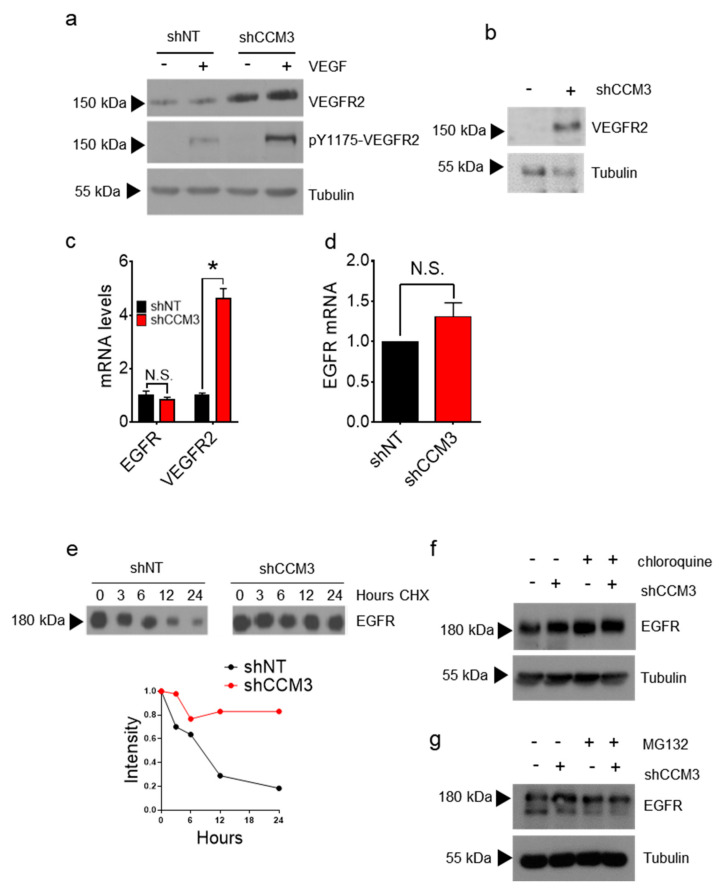
Upregulation of EGF family receptors in CCM3-deficient cells is postranscriptional; (**a**) VEGFR2 and phosphorylated VEGFR2 (pY1175) in shNT and shCCM3 HuVECs before and after VEGF stimulation; (**b**) Western blot of VEGFR2 in hCMEC/D3 cells; (**c**) mRNA levels of EGFR andVEGFR2 in HuVECs. * *p* < 0.05; (**d**) mRNA levels of EGFR in A549 cells; (**e**) EGFR in A549 cells transduced with shNT and shCCM3 shRNAs before and after treatment with the protein synthesis inhibitor cycloheximide. (**f**) EGFR Western blot in shNT and shCCM3 A549 cells treated or not with chloroquine. (**g**) Same as in (**f**) in cells treated or not with the proteasome inhibitor MG132.

As stated above, CCM deficiency affects the levels and function of VEGFR2, the major tyrosine kinase receptor in endothelial cells. We measured VEGFR2 levels in HuVECs transduced with shNT or shCCM3 RNAs. VEGFR2 was upregulated when CCM3 was inhibited, both in the absence and in the presence of VEGF (Figure 3a), although it had not shown up in the array. The same was true for hCMEC/D3 cells (Figure 3b). To explore the mechanisms of upregulation of the different proteins, we measured the mRNAs of VEGFR2 and EGFR in HuVECs (Figure 3c). Surprisingly, while the levels of VEGFR2 mRNA were clearly induced by lack of CCM3, EGFR mRNA was unaffected, suggesting that the effect of CCM3 on the levels of this protein was posttranslational. The mRNA levels of EGFR were also not induced in A549 cells (Figure 3d). To see if CCM3 affected the stability of EGFR, we looked at its levels in A549 cells treated with the protein synthesis inhibitor cycloheximide. EGFR half-life was prolonged in cells lacking CCM3 (Figure 3e). Moreover, chloroquine treatment resulted in higher levels of EGFR in both shNT and shCCM3 cells (Figure 3f), whereas treatment with the proteasome inhibitor MG132 did not (Figure 3g), suggesting that lysosomal degradation is the main route of EGFR degradation in these cells and that lack of CCM3 impairs it.

**Figure 4:**
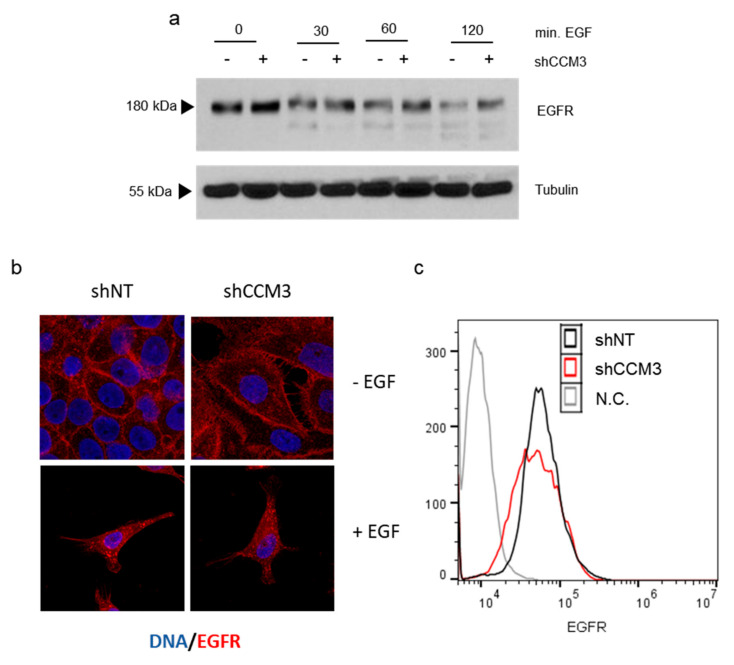
EGFR endocytosis is not inhibited in CCM3-deficient cells. (**a**) hCMEC/D3 endothelial cells transduced with shNT and shCCM3 shRNAs were serum deprived and treated with 100 ng/mL EGF for the indicated times, and EGFR levels were analyzed by Western blot; (**b**) A549 cells transduced with shNT and shCCM3 shRNAs were serum deprived and then treated or not with EGF 100 ng/mL previously. Then, cells were stained for immunofluorescence analysis of EGFR (red) expression and distribution. Images were captured at 63× and merged with DAPI (blue). (**c**) A549 cells transduced with shNT and shCCM3 shRNAs were serum deprived and the EGFR in their plasma membrane measured by flow cytometry.

**Figure 5:**
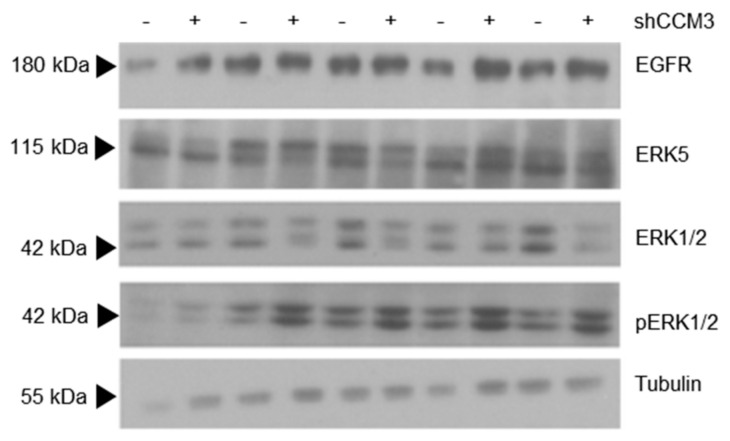
High levels of EGFR in CCM3-deficient cells results in enhanced phosphorylation of ERK1/2 after EGF treatment. A549 cells transduced with shNT and shCCM3 shRNAs were serum deprived and treated with 100 ng/mL EGF for the indicated times. EGFR, ERK5, ERK1/2 and pERK1/2 were analyzed by Western blot.

Lysosomal degradation of EGFR is a possible result of EGFR endocytosis after EGF stimulation, and CCM3 plays a role in endo and exocytosis [26,27,28]. To see if the difference in EGF receptor stability depended on EGF treatment, we serum-deprived cells and treated them with EGF to induce EGFR endocytosis and degradation. Both in hCMEC/D3 (Figure 4a), and A549 cells (Figure 5) EGFR levels were higher in shCCM3 cells even before EGF addition, suggesting that the effect of CCM3 on EGFR stability was not dependent on EGF. Moreover, we have not detected any defective endocytosis of EGFR in shCCM3 cells. EGFR is mainly in the plasma membrane in untreated cells, and is redistributed to intracellular vesicles after EGFR addition, independently of CCM3 status, as seen by immunofluorescence (Figure 4b). Furthermore, when the EGFR in the plasma membrane of serum-deprived cells was measured by flow cytometry, CCM3-deficient cells did not show a higher intensity than control cells (Figure 4c). We concluded that CCM3 did not affect EGFR endocytosis, before or after EGF addition.

Because EGFR is a druggable protein with well-characterized inhibitors, our next step was to see whether its higher levels in CCM3-deficient cells had a functional significance. One of the best characterized responses to EGFR stimulation by EGF is phosphorylation of Extracellular signal-regulated protein kinases 1 and 2 (ERK1/2), which we analyzed in endothelial cells untreated or treated with EGF for different times. Cells deficient for CCM3 had more phosphorylation of ERK1/2, in EGF-treated cells, which accompanied the expected effect on ERK5 phosphorylation, which also responded to EGF, as described [29] (Figure 5).

**Figure 6:**
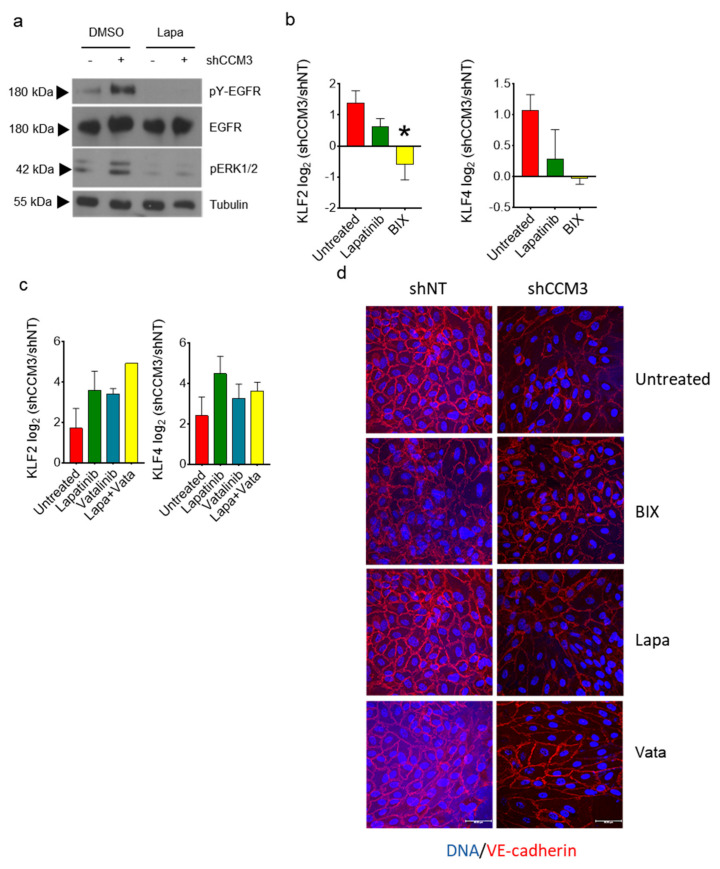
Inhibition of EGFR family receptors in CCM3-deficient cells does not affect overexpression of KLF transcription factors or VE cadherin distribution; (**a**) A549 cells transduced with shNT and shCCM3 shRNAs were treated or not with the EGFR family inhibitor Lapatinib 10 µM, and Western blots performed for the indicated proteins; (**b**) Difference of KLF2 (left) and KLF4 (right) expression in A549 cells transduced with shNT or with shCCM3 shRNAs, treated or not with Lapatinib or the MEK5/Erk5 inhibitor BIX02189. * *p* < 0.05; (**c**) Difference of KLF2 (left) and KLF4 (right) expression in hCMEC/D3 cells transduced with shNT or with shCCM3 shRNAs, treated or not with Lapatinib or the VEGFR2 inhibitor Vatalanib; (**d**) VE cadherin (red) distribution in hCMEC/D3 cells transduced with shNT or with shCCM3 shRNAs, treated or not with Lapatinib, BIX02189, or Vatalanib. Images are shown merged with DAPI (blue).

It is well established that CCM deficiency results in overactivation of the MEKK5/MEK3/ERK5 axis, and a resulting overexpression of the KLF2 and KLF4 transcription factors. To see if EGFR family members played a role in this deregulation in CCM3-deficient cells, we treated cells with Lapatinib, an inhibitor of the kinase activity of EGFR family proteins [30]. Lapatinib inhibited both EGFR and ERK1/2 phosphorylation in normal and CCM3-deficient A549 cells (Figure 6a). Despite this clear biochemical effect, Lapatinib did not consistently inhibit KLF2 or KLF4 overexpression in CCM3-deficient cells, showing at best a partial effect (Figure 6b). This was also true in endothelial cells, in which neither Lapatinib nor the VEGFR2-specific inhibitor vatalanib or the combination of both could revert the overexpression of KLF2 or KLF4 in shCCM3 cells (Figure 6c). Furthermore, the effect of CCM3 on the distribution of VE-cadherin was also not affected by treatment with either inhibitor or their combination (Figure 6d). We concluded that chemical inhibition of EGFR family members does not affect the canonical effect of CCM inhibition in endothelial or non-endothelial cells.

**Figure 7:**
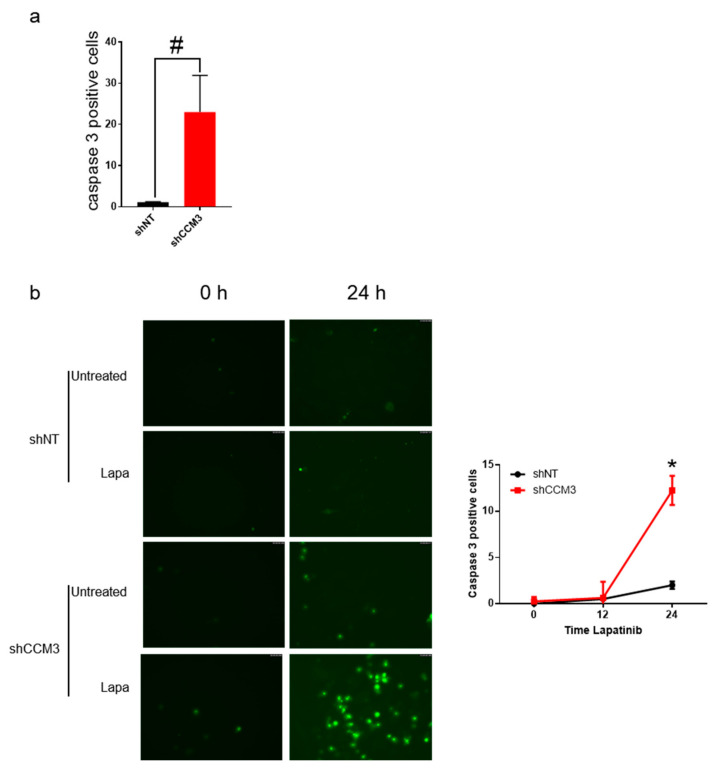
Deficiency of CCM3 makes endothelial cells more susceptible to inhibition of EGFR family receptors; (**a**) hCMEC/D3 cells transduced with shNT and shCCM3 shRNAs were treated with the EGFR family inhibitor Lapatinib for 24 h at 2 µM, apoptotic cells were detected. The number of cells with activated caspase 3/7 are represented. # *p* = 0.07. (**b**) HuVECs transduced with shNT and shCCM3 shRNAs were treated with 2 µM of the EGFR family inhibitor Lapatinib for 24 h and apoptotic cells were detected using the CellEvent™ Caspase-3/7 Green Detection Reagent (left panel). Number of caspase 3/7 positive cells at the indicated times were counted (right panel) * *p* < 0.05 vs. shNT.

Cancer cells that overexpress EGFR and/or ErbB2 are susceptible to their inhibition by Lapatinib. In fact, the main therapeutic effect of this drug is to induce cell growth arrest and death [31]. We wanted to know if Lapatinib could affect cell viability in a CCM3-dependent way. When hCMEC/D3 endothelial cells were treated with this drug at the clinically relevant dose of 2 µM [32,33], they had a higher tendency to enter apoptosis, as measured by incubation with a caspase 3/7 fluorogenic substrate (Figure 7a). Likewise, HuVECs depleted of CCM3 showed activated caspase 3/7 after 24 h incubation with Lapatinib only if CCM3-deficient (Figure 7b). Thus, we concluded that deficiency of CCM3 made endothelial cells susceptible to apoptosis by Lapatinib. While this is probably because of their overexpression of EGF receptor, formal proof of this would require genetic inhibition of EGFR.

## 4. Discussion

We show in this paper that CCM3 deficiency induces an overexpression of the EGF receptor, and a susceptibility of CCM3-deficient cells to inhibition of the EGFR family of receptors. 

Deregulation, usually overactivation, of VEGFR2 is a common alteration in cells deficient of a CCM gene. Lack of CCM1 induces VEGF deregulation and thus VEGFR2 activation, without higher levels of VEGFR2 protein [18,23], whereas CCM3-deficient cells have been reported to have a destabilized VEGFR2 [34], but more consistently to have enhanced VEGFR2 phosphorylation [24] and high VEGFR2 protein levels [25], which is in accordance with our results. 

In this article, we show that besides an upregulation of VEGFR2, CCM3-deficient cells have high levels of EGFR by a different mechanism: impaired degradation of the protein. Our results point to a defect in lysosomal degradation of the receptors in CCM3-deficient cells, despite which we have seen no apparent differences in the endocytosis of EGFR or its colocalization with lysosomal proteins, which suggests a specific defect in degradation itself, which affects the basal degradation of the protein, rather than its EGF-induced degradation. Alternatively, CCM3-deficient cells may have a defect in EGF receptor trafficking in a step different from the endocytic step. Although more experiments will be needed to ascertain this, it is intriguing that we and others had previously shown that inhibition of CCMs results in impaired autophagy [35,36], an effect that may be related to the one we are reporting here. It also agrees with one of the major phenotypic consequences of CCM and specifically of CCM3 deficiency, the altered handling of cellular membranes, which has been shown in several different models of CCM3 deficiency, as in C. elegans [26,37,38], D. melanogaster [39], zebrafish [40], and mammalian cells [27,28]. 

The deregulation of EGF receptor has a potentially important consequence: making cells susceptible to its inhibition, which could be a viable therapeutic strategy for cavernomas. We explored how this alteration on tyrosine kinase receptors relates with one of the main pathways affected by CCM3 loss. In our hands, EGF can stimulate ERK5 phosphorylation in serum-deprived cells, which is in accordance with previous reports [41]. Furthermore, we see that in early time points after stimulation ERK5 phosphorylation is higher in shCCM3 cells, an effect that we attribute to their higher receptor levels. However, the difference in ERK5 phosphorylation after EGF is transient, and it is lost in the last time points after treatment. Moreover, treatment with Lapatinib does not consistently inhibit the characteristic KLF2 and KLF4 overexpression of CCM3-deficient cells.

We also analyzed another potentially useful effect of tyrosine kinase inhibition: the ability to differentially affect viability of CCM3-deficient cells. We found that treatment with Lapatinib affects the viability of CCM3-deficient cells and not that of control cells. We propose that this differential effect is due to EGFR family inhibition, as we have used Lapatinib concentrations that are attained by the drug in several tissues in clinically relevant settings [32,33], together with the fact that Lapatinib is known to be a very specific inhibitor, targeting exclusively EGF receptor family members at these concentrations [30]. 

Curiously, similar observations have been made with other kinase inhibitors. Inhibition of VEGFR2 does not affect the overexpression of KLF2 or KLF4 after CCM2 loss [42], but it can partially prevent the development of cavernomas in CCM1-deficient animals [24]. Moreover, many of the drugs that have been shown effective in experimental models of CCM deficiency are known to inhibit tyrosine kinase receptors. It is the case for Ponatinib, an inhibitor of MEKK3/KLF signaling which prevents formation and progression of cerebral cavernous malformations [19]. Additionally, indirubin 3, which was identified as a CCM active molecule in a pharmacological screen [20], is known to inhibit both VEGFR2 and EGFR among other targets; and the same can be said of sorafenib, a multikinase inhibitor which reduces the effects of CCM1 loss in endothelial cells [43]. Thus, there is ample evidence that the use of compounds that inhibit receptor tyrosine kinases are a feasible strategy for CCM control.

Experiments in an animal model of CCM will be needed to know if Lapatinib administration is an effective strategy to treat cavernomas. This will also be essential to know if the strategy of killing the endothelial cells that form a cavernoma may have drawbacks, the most obvious being the possibility of lesion bleeding after endothelial death. Furthermore, most endothelial cells of patients with familial cavernomatosis are heterozygous for CCMs, and they may also be more susceptible to Lapatinib, a very unlikely possibility, as no haploinsufficient effect has been reported for CCMs to date.

While lesion bleeding would be a serious side effect that would compromise the use of this strategy, there are antecedents where endothelial cell death in vascular malformations do not lead to an increased risk of bleeding. The beta-blocker propranolol is regularly used to successfully treat infantile hemangiomas [44], and its mechanism of action has been shown to involve apoptotic killing of the hemangioma endothelial cells [45]. Despite this, no bleeding of these lesions has been described after treatment, and propranolol is the treatment of choice for this condition. Furthermore, as it happens with propranolol, killing of CCM deficient endothelial cells may not only prevent the development of new cavernomas, but also delete those already developed, which would be very useful in many clinical settings. 

## Supplementary Materials

The following are available online at https://www.mdpi.com/2227-9059/8/12/624/s1. Table S1: Sequences of oligonucleotide s used to generate lentiviral plasmids expressing shRNAs, Table S2, Sequences of oligonucleotides used for RT-qPCR, Table S3, Antibodies used for Western blot (WB) and immunofluorescence (IF).
