# Supplementary material for: High Levels of Receptor Tyrosine Kinases in CCM3-Deficient Cells Increase Their Susceptibility to Tyrosine Kinase Inhibition
Source: Biomedicines. 2020 Dec 17;8(12):624. doi: 10.3390/biomedicines8120624 (PMC7766026; doi:10.3390/biomedicines8120624)
Supplement: Supplementary file 1 [file biomedicines-08-00624-s001.pdf]

Sartages et al. Supplementary information:
Tables S1 to S3.

Table S1. Sequences of oligonucleotides used to generate lentiviral plasmids expressing shRNAs used in this article

**shRNA**		**Sequence**
ShNT	Sense	5'-CCGGCAACAAGATGAAGAGCACCAACTCGAGTTGGTGCTCTTCATCTTGTTGTTTTTG-3'
	Antisense	5'-AATTCAAAAACAACAAGATGAAGAGCACCAACTCGAGTTGGTGCTCTTCATCTTGTTG-3'
ShCCM3.1	Sense	5'-CCGGCCAGGATGTTGAATGGGATTACTCGAGTAATCCCATTCAACATCCTGGTTTTTG-3'
	Antisense	5'-AATTCAAAAACCAGGATGTTGAATGGGATTACTCGAGTAATCCCATTCAACATCCTGG-3'
ShCCM3.4	Sense	5'-CCGGCCAGATGAGATCAATGACAGACTCGAGTCTGTCATTGATCTCATCTGGTTTTTG-3'
	Antisense	5'-AATTCAAAAACCAGATGAGATCAATGACAGACTCGAGTCTGTCATTGATCTCATCTGG-3'

Table S2: Sequences of oligonucleotides used for RT-qPCR in this article.

Gene		Sequence
CCM3/PDCD10	Forward	5′-GCCCCTCTATGCAGTCATGTA-3′
	Reverse	5′-AGCCTTGATGAAAGCGGCTC-3′
VEGFR2	Forward	5′-GGCCCAATAATCAGAGTGGCA-3′
	Reverse	5′-CCAGTGTCATTTCCGATCACTTT-3′
GAPDH	Forward	5′-GGAGCGAGATCCCTCCAAAAT-3′
	Reverse	5′-GGCTGTTGTCATACTTCTCATGG-3′
β-ACTIN	Forward	5′-CATGTACGTTGCTATCCAGGC-3′
	Reverse	5′-CTCCTTAATGTCACGCACGAT-3′
EGFR	Forward	5′-AGGCACGAGTAACAAGCTCAC-3′
	Reverse	5′-ATGAGGACATAACCAGCCACC-3′
ErbB2/HER2	Forward	5′-TGCAGGGAAACCTGGAACTC-3′
	Reverse	5′-ACAGGGGTGGTATTGTTCAGC-3′
KLF4	Forward	5′-CCCACATGAAGCGACTTCCC-3′
	Reverse	5′-CAGGTCCAGGAGATCGTTGAA-3′
KLF2	Forward	5′-CTACACCAAGAGTTCGCATCTG-3′
	Reverse	5′-CCGTGTGCTTTCGGTAGTG-3′

Table S3. Antibodies used for western blot (WB) and immunofluorescence (IF) in this article.

Antigen	Host	Clonality	Company	Catalogue	Application	Dilution
CCM3	mouse	monoclonal	Proteintech Group	#66440	WB	1:1000
ErbB2	rabbit	monoclonal	Abcam	#ab134182	WB	1:1000
α-tubulin clone B5-1-2	mouse	monoclonal	Sigma	#T5168	WB	1:4000
EGFR	rabbit	monoclonal	Cell Signalling Technology	#4267	WB	1:1000
EGFR	rabbit	monoclonal	Cell Signalling Technology	#4267	IF	?
phospho-Tyr1148-EGFR	rabbit	polyclonal	Cell Signalling Technology	#4404	WB	1:1000
GAPDH	mouse	monoclonal	Santa Cruz Biotechnology	#sc-47724	WB	1:1000
VEGFR2	rabbit	monoclonal	Cell Signalling Technology	#2479	WB	1:1000
phospho- Tyr1175-VEGFR2	rabbit	monoclonal	Cell Signalling Technology	#2478	WB	1:1000
Erk5	rabbit	polyclonal	Cell Signalling Technology	#3372	WB	1:500
phospho-Thr218/Tyr220-Erk5	rabbit	polyclonal	Cell Signalling Technology	#3371	WB	1:1000
Erk1/2	rabbit	monoclonal	Cell Signalling Technology	#4695	WB	1:1000
phospho-Thr202/Tyr204-Erk1/2	rabbit	monoclonal	Cell Signalling Technology	#4370	WB	1:1000
VE-Cadherin	mouse	monoclonal	Abcam	#ab7047	IF	1:150
